# Cannabinoid Receptor 2 Signaling in Neurodegenerative Disorders: From Pathogenesis to a Promising Therapeutic Target

**DOI:** 10.3389/fnins.2017.00030

**Published:** 2017-02-02

**Authors:** Tommaso Cassano, Silvio Calcagnini, Lorenzo Pace, Federico De Marco, Adele Romano, Silvana Gaetani

**Affiliations:** ^1^Department of Clinical and Experimental Medicine, University of FoggiaFoggia, Italy; ^2^Department of Physiology and Pharmacology, Sapienza University of RomeRome, Italy; ^3^Laboratory of Virology, The Regina Elena National Cancer InstituteIRCCS, Rome, Italy

**Keywords:** Alzheimer's disease, Parkinson's disease, neuroprotection, neuroinflammation, microglia, astrocytes

## Abstract

As a consequence of an increasingly aging population, the number of people affected by neurodegenerative disorders, such as Alzheimer's disease, Parkinson's disease and Huntington's disease, is rapidly increasing. Although the etiology of these diseases has not been completely defined, common molecular mechanisms including neuroinflammation, excitotoxicity and mitochondrial dysfunction have been confirmed and can be targeted therapeutically. Moreover, recent studies have shown that endogenous cannabinoid signaling plays a number of modulatory roles throughout the central nervous system (CNS), including the neuroinflammation and neurogenesis. In particular, the up-regulation of type-2 cannabinoid (CB2) receptors has been found in a number of neurodegenerative disorders. Thus, the modulation of CB2 receptor signaling may represent a promising therapeutic target with minimal psychotropic effects that can be used to modulate endocannabinoid-based therapeutic approaches and to reduce neuronal degeneration. For these reasons this review will focus on the CB2 receptor as a promising pharmacological target in a number of neurodegenerative diseases.

## Introduction

The field of cannabinoid (CB) research has flourished over the past decade and has brought to light diverse functions of the CB system in normal and pathological conditions (D'Addario et al., 2014; Bonnet and Marchalant, 2015). In fact, several studies have demonstrated that endocannabinoid (eCB) system plays significant roles in many biological processes, including neurogenesis, synaptic plasticity, emotional regulation and stress responsiveness (Lu and Mackie, 2015).

The eCB system consists of eCBs, cannabinoid receptors and enzymes involved in the synthesis and degradation of endogenous ligands (Lu and Mackie, 2015).

The eCBs are endogenous lipids that engage CB receptors, affecting behavior in a fashion that at least partially recapitulates the effects produced by the psychoactive components of cannabis, most notably (2)-trans-Δ9-tetrahydrocannabinol (THC) (Mechoulam and Gaoni, 1965; Mechoulam, 1970). The two best-characterized eCBs are N-arachidonoylethanolamide (anandamide, AEA) (Devane et al., 1992) and 2-arachidonoylglycerol (2-AG) (Mechoulam et al., 1995; Sugiura et al., 1995). Both eCBs are synthetized at the post-synaptic terminal from membrane lipid precursors in response to high intracellular calcium concentration (Howlett et al., 2002). Thus, eCBs act as retrograde messengers to depress transmitter release from presynaptic terminals (Freund et al., 2003; Fagan and Campbell, 2014).

AEA and 2-AG possess specific pharmacological properties, are engaged in different forms of synaptic plasticity and modulate different behavioral functions (Mechoulam and Parker, 2013).

The CB type 1 (CB1) and type 2 (CB2) receptors are coupled to G-protein, and their signal transduction is mediated by the inhibition of adenylyl cyclases and voltage-gated calcium channels (e.g., N-type, P/Q-type and L-type calcium currents), and by the activation of mitogen-activated protein kinases (MAPK) and inwardly rectifying potassium channels (Howlett et al., 2002; Lu and Mackie, 2015). AEA is a high affinity, CB1-selective partial agonist, whereas 2-AG is a moderate affinity, CB1/CB2 full agonist (Sugiura et al., 2000). AEA activates also peroxisome proliferator-activated receptors-alpha and transient receptor potential vannilloid-1 channels (Maccarrone et al., 2010). In humans, CB1 is localized preferentially in the terminals of central and peripheral neurons and glial cells, where it regulates neurotransmitter release and psychoactivity (Egertová et al., 2003; Sánchez and García-Merino, 2012). As far as peripheral tissues, CB1 is also expressed in heart, uterus, testis, liver and small intestine, as well as in immune cells (Maccarrone et al., 2001; Nong et al., 2001; Klein et al., 2003) and adipose tissue (Spoto et al., 2006).

CB2 was dubbed the “peripheral cannabinoid receptor” as a result of *in situ* hybridization study that showed high CB2 mRNA expression in spleen, whereas no expression was observed in the brain (Shire et al., 1996; Griffin et al., 2000; Brown et al., 2002). Besides the cells of the immune and hematopoietic systems (e.g., leukocytes, spleen and tonsils), CB2 receptors were found also in other peripheral organs, such as muscle, liver, intestine and testis (Liu et al., 2009). However, CB2 receptor can be also detected in the central nervous system (CNS) (albeit at a lower expression level than CB1receptors) (Núñez et al., 2004; Van Sickle et al., 2005), where its expression is significantly increased following a number of stressful conditions (Viscomi et al., 2009). In particular, CB2 receptor expression is found in neurons within the brainstem, microglia and astrocytes only after specific insults (e.g., neuroinflammation), whereas it cannot be detected in resting microglia (Van Sickle et al., 2005; Núñez et al., 2008; Cabral and Griffin-Thomas, 2009).

In the last decade, increasing evidence has shown that CB receptors may act as CB1-CB2 receptor heteromers in the brain (Callén et al., 2012). In fact, the expression of CB1-CB2 receptor heteromers was determined in a variety of brain regions, such as the nucleus accumbens, pineal gland and globus pallidus (Callén et al., 2012). Due to this tight functional interaction between CB receptors, the response to molecules acting as agonists or antagonists may be different when a CB receptor is engaged in heteroreceptor complexes. Although the clinical relevance of this phenomenon is not entirely clear, additional studies are needed in order to shed further light on this important functional interaction.

eCBs after their actions are rapidly eliminated by cellular uptake and enzymatic hydrolysis. To this regard, AEA is mainly inactivated by fatty acid amide hydrolase (FAAH) (Cravatt et al., 1996; Dinh et al., 2002), whereas 2-AG is predominantly catalyzed by monoacylglycerol lipase (Dinh et al., 2002).

As previously reported, CB1 receptor expression is abundant in the CNS, where it seems to mediate the psychoactive effects of cannabis (Mackie, 2005). Therefore, the scarcity of CNS CB2 receptors makes CB2 selective drugs attractive as therapeutics as they would presumably invoke minimal psychoactive responses. In support of this hypothesis, CB2 knockout mice demonstrated typical behavioral responses to THC but lost their normal immune responsiveness to THC (Buckley et al., 2000). CB2 levels are also increased under certain conditions and disease states further adding to its attractiveness as a potential therapeutic target (Zhang et al., 2003; Wotherspoon et al., 2005; Yiangou et al., 2006).

Therefore, we will review the role of eCB system in two chronic neurodegenerative diseases, in which the neuroprotective effects following CB receptors modulation have been reported in different studies. Specifically, we will focus on the role of CB2 receptors and their agonists, as potential therapeutical targets in Alzheimer's disease (AD) and Parkinson's disease (PD).

## Role of CB2 receptor in the neurodegeneration and neuroprotection

Recently, much research has paid attention to the neuroprotective effects of compounds targeting the eCB system. In particular, these studies have focused on identifying molecular targets within the eCB system that may lead to neuroprotection against the most prevalent neurodegenerative disorders (Fernández-Ruiz et al., 2010, 2015).

One of the most important features of CBs as potential neuroprotectants is their broad-spectrum of activity. This aspect is particularly important in neurodegenerative diseases since declines in neural function are likely due to the concerted involvement of different insults including protein misfolding, neuroinflammation, excitotoxicity, oxidative stress and mitochondrial dysfunction (Serviddio et al., 2011; Cassano et al., 2012, 2016; Aureli et al., 2014). All these pathological processes appear to be modulated by the eCB signaling system. In fact, during aging and neuroinflammation (or when both are present together) there is a widespread disruption of brain tissue homeostasis that involves eCB signaling, and this contributes to specific dysfunctions in cell function.

Although the CNS is considered a relatively immune-privileged tissue, it is able to initiate an endogenous immune response. To this regard, astrocytes and microglia are the main innate immune response effectors in brain parenchyma (Halliday and Stevens, 2011).

The most extensively studied mechanism of neuroprotection includes the anti-inflammatory effects of the CB2 receptors, in which CB2 protects the brain by restraining inflammatory processes (Benito et al., 2008; Cabral and Griffin-Thomas, 2009). In particular, CB2 receptor activation modulates the release of cytokines, protein molecules responsible for the regulation of immune function and inflammatory responses (Mecha et al., 2016; Turcotte et al., 2016). Differently, the CB1 receptor has been implicated in protection against cell death induced by an overstimulation of excitatory receptors and concurrent calcium release, also known as excitotoxicity (Vendel and de Lange, 2014). CB receptors, therefore, may have an impact on neurodegenerative diseases through two main ways, restraining exitotoxic and immunological processes (Di Iorio et al., 2013).

Moreover, it has been demonstrated that changes in the expression of CB receptors may be time-dependent and could occur both in the brain and peripheral tissues at different stages of the neurodegenerative process (Bedse et al., 2014, 2015; Di Marzo et al., 2015). For this reason, targeting the CB receptors for therapeutic benefit needs more caution. To this regard, CB1 activity was higher at earlier AD stages in limited hippocampal areas and internal layers of the frontal cortex, but a decrease was observed during the advanced stages (Lastres-Becker et al., 2001; Manuel et al., 2014; Rodríguez-Cueto et al., 2014). The increased CB1 receptor activity during the initial stages of AD may indicate neuroprotective action mediated by eCBs in response to initial neuronal damage.

However, CB1 receptors are not usually considered as realistic targets for neuroprotection, because during neurodegenerative processes it has been described a progressive loss of specific populations of neurons that express CB1 receptors (Ramírez et al., 2005; Solas et al., 2013). In line with these results, our group (Bedse et al., 2014), but also Kalifa et al. (2011) reported a decrease in CB1 protein expression in transgenic mice models of AD.

In contrast, CB2 receptors are generally less expressed in the neurons of healthy brains, but their expression increases dramatically in reactive microglia and activated astrocytes during neuroinflammation (Stella, 2010; Di Marzo et al., 2015; Fernández-Ruiz et al., 2015). Therefore, the CB2 receptors have the potential to restrain the inflammatory processes that contribute to the declines in neural function occurring in a number of neurodegenerative disorders.

## CB2 receptors and Alzheimer's disease

AD is a devastating neurodegenerative disease leading to progressive cognitive dysfunction. The iconic hallmarks of AD are Aβ plaques, neurofibrillary tangles (NFTs) and a deficiency in cholinergic neurotransmission. It is widely accepted that the deposition of Aβ initiates an inflammatory process leading to neurodegeneration (McGeer et al., 2000; Walsh and Selkoe, 2004). Microglial cells are the resident CNS phagocytes of the immune system that mediate inflammatory responses to pathogens and injury by inducing release of pro-inflammatory cytokines including interleukin (IL)-1β, IL-6, and tumor necrosis factor-α (TNF-α). IL-1β and TNF-α are considered as primary cytokines responsible for chronic inflammation in AD (Sastre et al., 2006). Microglia-derived pro-inflammatory cytokines, in turn, aggravate and propagate inflammation throughout the brain. In fact, IL-1β released from microglia can induce the upregulation of nuclear factor-kappa B (NFκB), MAPK, and Jun-N-terminal kinase (JNK) signaling in neurons and astrocytes, leading to increased inflammatory process and tau phosphorylation, respectively (Sastre et al., 2006; Munoz and Ammit, 2010). Additionally, Aβ oligomers can induce production of inducible nitric oxide synthase (iNOS), nitric oxide (NO), and TNF-α in astrocytes (White et al., 2005). NO secreted from astrocytes induces abnormal tau hyperphosphorylation in neurons, which prompts an accumulation of NFTs in axons, leading to a disruption of synaptic plasticity and neuronal death (Duan et al., 2012). Moreover, the activation of toll-like receptors (TLR; e.g., TLR-4), involved in pathogen recognition and activation of innate immunity, can also activate the MAPK and NFκB pathways, as well as members of the caspase family responsible for hyperphosphorylation of tau (Churcher, 2006; Reed-Geaghan et al., 2009; Rohn, 2010; Arroyo et al., 2011). Activation of these signaling cascades in neurons could further inhibit synaptic plasticity.

Support for the involvement of the CB2 receptors in AD pathology is provided by a number of preclinical and human studies. In particular, post-mortem brains from patients with AD have shown that CB2 receptors are upregulated in cells that are associated with Aβ-enriched neuritic plaques (Benito et al., 2003; Ramírez et al., 2005; Grünblatt et al., 2009; Halleskog et al., 2011; Mulder et al., 2011; Solas et al., 2013). Apart from human studies, transgenic models of AD have also revealed overexpression of CB2 receptors in brain areas affected by AD-pathology (Horti et al., 2010). Increased CB2 mRNA in peripheral blood has been suggested as a peripheral biomarker for the early diagnosis of AD (Grünblatt et al., 2009). Moreover, an increase in CB2 receptors was also observed in rats and C6 astroglioma cells pre-treated with Aβ42 (Esposito et al., 2007).

All these effects may be counteracted by the activation of CB2 receptors, and mechanistic insights of the beneficial effects provided by CB2 receptor stimulation in AD has been provided (Ehrhart et al., 2005; Ramírez et al., 2005; Sheng et al., 2005; Chen et al., 2010; Fakhfouri et al., 2012; Martin-Moreno et al., 2012) (Table 1). In particular, the CB2 agonist, JWH-015, significantly attenuated CD40-mediated inhibition of microglial phagocytosis of Aβ42 by interfering with the Janus kinase/Signal transducer and activator of transcription 1 (JAK/STAT1) pathway (Benveniste et al., 2004; Ehrhart et al., 2005). Interestingly, CP55940 (CB1/CB2 full agonist) and JWH-015 treatment significantly reduced the interferon-gamma- (IFN-γ)-induced CD40 expression in microglial cells (Ehrhart et al., 2005).

**Table 1:** **CB2 receptor agonists and their beneficial effects in neurodegenerative diseases (AD and PD)**.

**Subjects**	**CB2 agonists**	**Effects and mechanisms involved**	**References**
**ALZHEIMER's DISEASE (AD)**			
IFN-γ-activated microglial cells (Aβ42 insult)	JWH-015	↓ CD40 expression induced by IFN-γ;	Ehrhart et al., 2005
		↓ JAK/STAT1 phosphorylation;	
	CP55940	↑ phagocytosis of Aβ42;	
		↓ TNF-α and NO release.	
Microglial cells (Aβ insult)	WIN55,212-2	↓ Microglial cell Aβ induced activation;	Ramírez et al., 2005
	JWH-133	↓ TNF-α release.	
Aβ-induced hippocampal neurodegeneration in adult rats	WIN55,212-2	↑ Memory functions;	Fakhfouri et al., 2012
		↓ TNF-α release;	
		↓ caspases-3 activation;	
		↓ nuclear NFκB levels.	
IL-1β-activated human fetal astrocytes	WIN55,212-2	↓ iNOS expression;	Sheng et al., 2005
		↓ TNF-α and NO release;	
		↓ chemokines release (CXCL10, CCL2, CCL5).	
Tg2576 mice	WIN55,212-2	↓ cognitive impairments;	Martin-Moreno et al., 2012
	JWH-133	↓ microglial activation;	
		↓ COX-2 expression;	
		↓ TNF-α release;	
		↓ cortical Aβ deposition.	
**PARKINSON's DISEASE (PD)**			
MPTP-lesioned mice	WIN55,212-2	↓ microglial activation;	Price et al., 2009
	JWH-015	↓ degeneration of nigro-striatal DA neurons;	
		↓ MPTP-induced motor deficits;	
		↑ dopamine and 3,4-dihydroxyphenylacetic acid levels in SNc and dorsal striatum;	
		↑ TH^+^ neurons in the SNc.	
IFN-γ-activated microglial cells	JWH-015	↓ CD40 expression induced by IFN-γ;	Ehrhart et al., 2005
	CP55940	↓ JAK/STAT1 phosphorylation;	
		↓ TNF-α and NO release.	
Human microglial cells (from temporal lobe)	JWH-015	↑ neuroprotective effects;	Klegeris et al., 2003
		↓ TNF-α and IL-1β release (JWH-015);	
	BML-190	↑ TNF-α release	
		(BML-190).	
Primary astrocyte cultures from 1 day-old CD1 mouse brains (LPS insult)	CP55940	↓ iNOS expression;	Molina-Holgado et al., 2002
	HU-210	↓ NO release.	
Primary glial cells and cerebrocortical neurons from 1 day-old mouse brains (LPS insult)	CP55940	↑ IL-1ra and NO release (primary glial cells);	Molina-Holgado et al., 2003
	HU-210	↑ neuroprotective effects.	
LPS-lesioned rats	HU-308	↑ neuroprotective effects;	García et al., 2011
		↑ TH^+^ neurons in the substantia nigra.	
LPS-lesioned mice	HU-308	↓ CD68, iNOS, TNF-α and IL-1β expression in the striatum;	Gómez-Gálvez et al., 2016
		↑ TH^+^ neurons in the substantia nigra;	
		↓ TNF-α expression in the substantia nigra.	
*Drosophila melanogaster* (paraquat insult)	CP55940	↑ fly survival and locomotor activities;	Jimenez-Del-Rio et al., 2008
		↓ activation of JNK signaling.	
6-OHDA-lesioned rats	HU-308	↓ dopamine depletion in caudate putamen;	García-Arencibia et al., 2007
		↑ TH activity in caudate putamen (HU-308);	
	WIN55,212-2	= TH-mRNA levels in the substantia nigra	
		(HU-308).	

*IL-1ra, endogenous IL-1 receptor antagonist*.

Ramírez and colleagues demonstrated the effects of CB receptor agonists on microglial activation (Ramírez et al., 2005). Authors studied *in vitro* the effects of WIN55,212-2, the mixed CB1/CB2 agonist devoid of antioxidant properties (Howlett et al., 2002; Marsicano et al., 2002), HU-210 and JWH-133, respectively CB1 and CB2 selective agonist, in Aβ-induced microglial cells (Ramírez et al., 2005). As expected, Aβ peptide activated microglial cells and this was associated with increased mitochondrial activity, TNF-α release, cellular morphological changes and secretion of pro-inflammatory cytokines. Cannabinoid treatments prevented the enhancement of TNF-α release and counteracted Aβ-mediated activation of microglia (Ramírez et al., 2005).

The protective properties of WIN55,212-2 were also demonstrated in Aβ-induced neurodegeneration in rat hippocampus. WIN55,212-2 significantly improved memory functions and decreased the elevated levels of neuroinflammatory markers like TNF-α, activated caspase-3, and nuclear NFκB. The use of antagonists confirmed that these neuroprotective effects of WIN55,212-2 were partially mediated by CB1 and CB2 receptors (Fakhfouri et al., 2012). Moreover, WIN55,212-2, through CB2 receptors, inhibited iNOS and NO production, the release of chemokines (CXCL10, CCL2, and CCL5) and TNF-α from IL-1β-activated human fetal astrocytes (Sheng et al., 2005). The CB1 and CB2 receptor-specific antagonists SR141716A (Micale et al., 2013) and SR144528 (Saito et al., 2010), respectively, partially blocked this suppressive effect, which suggests the involvement of both receptors (Sheng et al., 2005).

**Figure 1:**
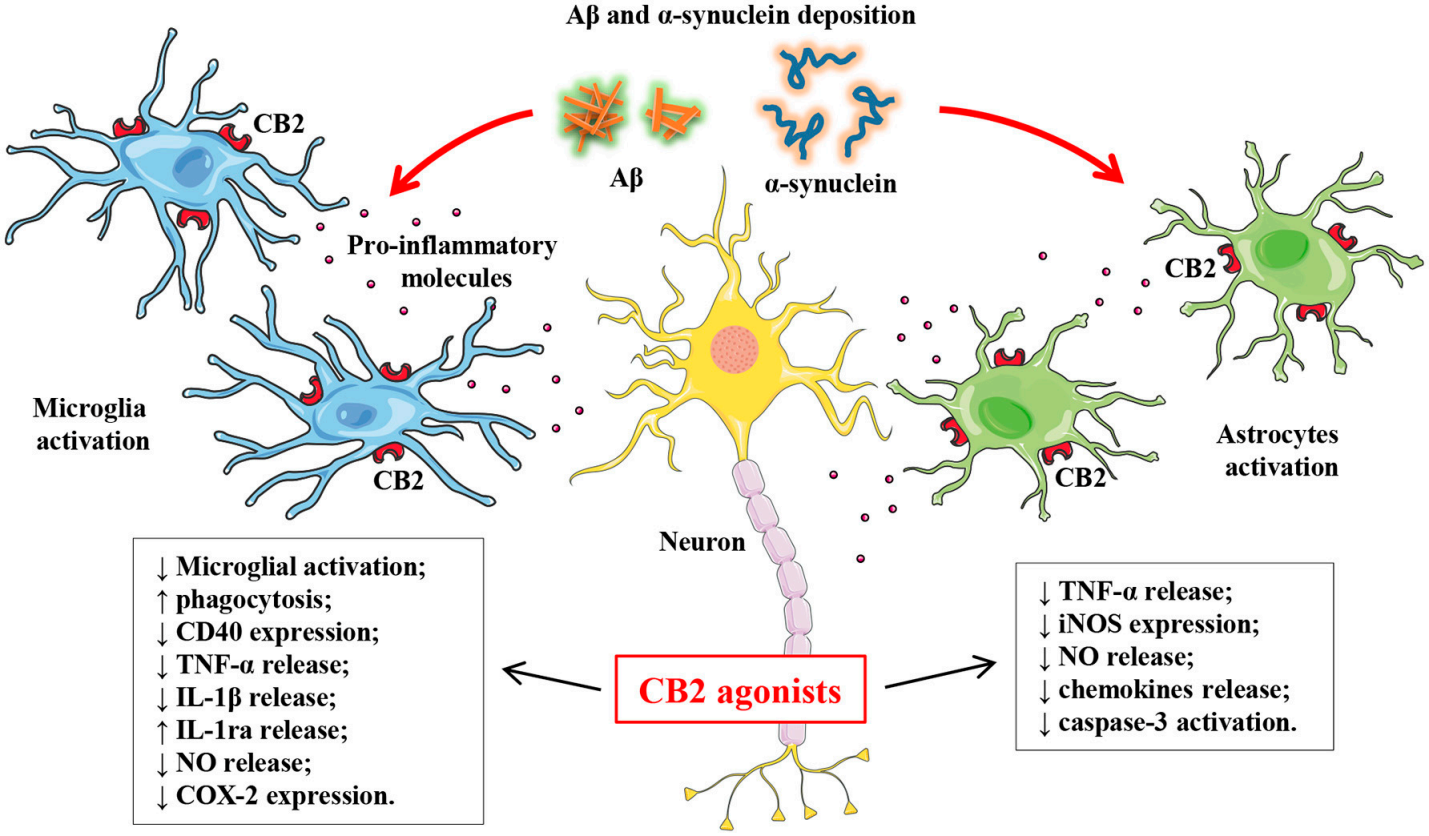
**Schematic representation of anti-inflammatory and neuroprotective actions of CB2 agonists in AD and PD**. AD and PD are characterized respectively by the deposition of Aβ and α-synuclein proteins which in turn are directly or indirectly involved in microglial and astrocytic activation. This activation of microglia and astrocytes triggers a neuroinflammatory and immune response which contributes to the progression of AD and PD. The pharmacological activation of microglial and astrocytic CB2 cannabinoid receptors with CB2 agonists is a promising therapeutic approach because it promotes anti-inflammatory and neuroprotective effects such as the suppression of pro-inflammatory cytokine release and an increases in anti-inflammatory molecules.

Furthermore, the effects of cannabinoids were studied in transgenic murine models of AD treated chronically with WIN55,212-2 or JWH-133, a potent selective CB2 receptor agonist (Martin-Moreno et al., 2012). JWH-133 was able to reduce cognitive impairments and decrease microglial activation in Tg2576 mice, while WIN55,212-2 was ineffective. Moreover, both cannabinoids significantly reduced the increase of COX-2, TNF-α, and cortical Aβ levels, suggesting a critical role of CB2 in inflammatory processes in AD (Martin-Moreno et al., 2012) (Figure 1).

From this scenario has emerged that the pleiotropic effects of CB2 agonists and the growing number of preclinical effects on AD rodent models should engage the interest of the research community and be seen as a valuable potential alternative treatment strategy to slow the progression and reduce the symptoms of cognitive decline in AD.

## CB2 receptors and Parkinson's disease

PD, the second most common neurodegenerative disease, is characterized by the progressive loss of dopaminergic neurons primarily in the *substantia nigra* (SN) affecting the circuits of the basal ganglia resulting in bradykinesia, rigidity and tremors (de Lau and Breteler, 2006; Branchi et al., 2008, 2010; Bartels and Leenders, 2009). Current treatments include dopaminergic replacement therapies, which do alleviate some of the symptoms but there are no available therapies that reverse any of the underlying pathological mechanisms (Calne et al., 2005; Trapani et al., 2011; Denora et al., 2012; Di Gioia et al., 2015).

Moreover, there is an urgent need for a novel intervention aimed at the prevention of dyskinesia induced by long-term treatment with levodopa. To this regard, a randomized double-blind crossover study showed that cannabis, which contains more than 70 different cannabinoids (Mechoulam, 2005), failed to demonstrate efficacy in treating dyskinetic patients with PD (Carroll et al., 2004). Unfortunately, the latter study suffered from methodological issues such as including small numbers of patients, and having inadequate power to detect a small change in dyskinesia.

PD is accompanied by multiple changes in the brain that underlie the progression of the disease. In this context, inflammation is an important pathogenic factor in sporadic PD, where it is thought to disable or kill dopaminergic neurons of the SN, which contributes to the dopaminergic denervation of the striatum.

The involvement of inflammation in PD has been initially investigated by McGeer et al. (1988), who showed microglia activation in the SN of patients at post-mortem. Afterwards, more evidence has accumulated that highlights the role of the neuroinflammation in the pathogenesis of PD. In line with this, *in vivo* studies using structural brain imaging have demonstrated in the nigrostriatal system of PD patients the presence of activated microglia and an increase of proinflammatory cytokines, including TNF-α, IL-1β, IL-2, IL-4, and IL-6 (Ouchi et al., 2005; Gerhard et al., 2006; Taylor et al., 2013).

α-synuclein (α-syn), the major component of Lewy bodies, is another pre-disposing element in PD etiology (Spillantini et al., 1998; Aureli et al., 2014). Missense mutations in the α-syn gene have been identified to cause autosomal dominant familial PD (Polymeropoulos et al., 1997; Krüger et al., 1998; Zarranz et al., 2004). Several lines of evidence suggest that α-syn may play an important role in the microglia-mediated inflammatory response in PD (Zhang et al., 2005; Austin et al., 2006; Reynolds et al., 2007, 2008; Thomas et al., 2007; Gao et al., 2008; Klegeris et al., 2008; Aureli et al., 2014). It is believed that genetic and environmental factors may initiate the neurodegeneration, which is further sustained or exacerbated by neuroinflammation leading to a “self-sustaining” process (Tansey and Goldberg, 2010). Therefore, effective anti-inflammatory intervention may arrest this cyclical process and counteract the neuroinflammation-induced neuronal degeneration.

Recently, in post-mortem study it has been demonstrated that PD patients showed elevated expression of CB2 receptors in microglial cells of SN (Gómez-Gálvez et al., 2016). In this context, as for AD, converging evidence indicates that CB2 receptor may represent a promising anti-inflammatory target in PD (Figure 1, Table 1). This hypothesis comes from numerous studies where the pharmacological activation of microglial CB2 receptors produced a reduction of microglial activation and functional deficits in the 1-methyl-4-phenyl-1,2,3,6-tetrahydropyridine (MPTP) mouse model of PD (Price et al., 2009), the suppression of pro-inflammatory cytokine release (Molina-Holgado et al., 2002; Klegeris et al., 2003; Ehrhart et al., 2005), and an increase in anti-inflammatory cytokines (Molina-Holgado et al., 2003). Moreover, CB2 receptor–deficient mice have shown an exacerbation of the PD pathology with increased microglial activation, neural alterations and functional deficits. Similar effects were also observed in other models of PD, such as MPTP-lesioned and lipopolysaccharide- (LPS)-injected mice (Price et al., 2009; García et al., 2011; Gómez-Gálvez et al., 2016). Moreover, the genetic ablation of the CB2 receptor protects against nigro-striatal damage following 6-hydroxydopamine (6-OHDA) lesion in mice (Ternianov et al., 2012).

Neuroprotection has been provided by synthetic cannabinoids such as the CP55,940, CB1/CB2 full agonist (Jimenez-Del-Rio et al., 2008), which acts through CB receptor-independent mechanisms, and involves the control of endogenous antioxidant defenses. In particular, authors found that CP55,940 protects Drosophila melanogaster mutants which lack CB receptors (McPartland et al., 2001; Elphick and Egertová, 2005), and alleviates the toxicity induced by paraquat (Jimenez-Del-Rio et al., 2008). The latter effect was exerted by the inactivation of JNK signaling and CB receptors were not involved (Jimenez-Del-Rio et al., 2008). Other findings concerning the possible off-target effects of CB agonists were obtained also from *in vivo* studies, in which mice genetically deleted of CB receptors were treated with molecules targeting “non-cannabinoids” receptors (see for review Pertwee et al., 2010).

Selective CB2 receptor agonists induced gains of function in MPTP-lesioned mice (Price et al., 2009) and LPS-injected mice (García et al., 2011), but not in 6-OHDA-lesioned rats (García-Arencibia et al., 2007). The lack of effects of CB2 agonists may be due to a lower inflammatory response induced by 6-OHDA compared to that caused by LPS and MPTP (Price et al., 2009; García et al., 2011). In particular, HU-308, the selective CB2 agonist, reversed the LPS-induced reduction of tyrosine hydroxylase positive (TH^+^) neurons and the elevation of CD68 immunostaining in the striatum, which identifies activated microglia and infiltrated peripheral macrophages. Moreover, authors found that HU-308 significantly reduced increases in striatal iNOS gene expression following an LPS insult (Gómez-Gálvez et al., 2016). In line with these results, García and colleagues found that HU-308 preserved TH^+^ neurons in the SN of LPS-injected mice (García et al., 2011).

A comprehensive study conducted by Price et al. (2009) demonstrated that the chronic treatment with the non-selective CB receptor agonist WIN55,212-2 protected against MPTP-induced loss of TH^+^ neurons in the SN pars compacta (SNc), independently of CB1 receptor activation. In fact, the authors found that WIN55,212-2 was still able to protect TH^+^ neurons from MPTP-lesioned CB1 receptor–deficient mice. Moreover, WIN55,212-2 increased the levels of dopamine and 3,4-dihydroxyphenylacetic acid in the SNc and dorsal striatum of MPTP-lesioned mice and reversed MPTP-associated motor deficits. WIN55,212-2 or JWH015, agonist of CB2 receptor, reduced MPTP-induced microglial infiltration. The suppressive effect of WIN55,212-2 and JWH015 on microglia was due specifically to CB2 activation as it was reversed by the CB2 antagonist JTE (Price et al., 2009).

Unlike targeting CB2 receptor signaling, the activation of CB1 receptors may cause hypokinetic side effects that could aggravate the major symptoms of PD, such as bradykinesia (García-Arencibia et al., 2009). Therefore, the modulation of CB1 receptors seems not to be a promising target for therapeutic intervention in PD. However, CB1 activation may alleviate the levodopa-induced dyskinesia, a motor complication resulting from long-term use of levodopa (Morgese et al., 2007, 2009).

Taken together these results demonstrate that CB2 receptors play an important role in the pathophysiology of PD and that their activation with selective agonist may lead to neuroprotective effect in the neurodegenerative processes of PD.

## Conclusions

Several lines of evidence suggest a major involvement of inflammation in the neurodegenerative process and therapeutic intervention strategies limiting the inflammatory responses secondary to microglial activation have been proposed by different authors based on many preclinical researches. Furthermore, recent approaches to the development of novel therapeutic strategies for neurodegenerative diseases have focused on their neuroprotective properties rather than concentrating on palliating symptoms of the diseases. Because cannabinoids possess both anti-inflammatory and neuroprotective actions, the use of CB2 receptor agonists offers an interesting, novel and promising therapeutic approach for a range of neurodegenerative disorders.

Moreover, modulation of CB2 receptor function has considerable therapeutic advantages over the modulation of the CB1 receptor, since the selective expression of CB2 receptors on the microglial cells provides a highly specialized target, without the psychoactivity due to CB1 activation. Although more studies are necessary to dissect the molecular mechanisms which lead to changes in CB2 receptor expression in AD and PD, these studies suggest that CB2 receptors may be key regulators of neuroinflammation and may be successfully targeted by therapeutic intervention.

## Author contributions

All authors have contributed to the writing, design and preparation of figures. Coordination of efforts has been carried out by the senior authors (TC and SG) of the three participating laboratories.

## Funding

This study was supported by Grant PRIN 2012 (to SG) (PRIN: 2012JTX3KL_002) and the Post-Doctoral fellowship of Dr Adele Romano (FIR: RBFR12DELS_003).

### Conflict of interest statement

The authors declare that the research was conducted in the absence of any commercial or financial relationships that could be construed as a potential conflict of interest.
